# Covalent Porphyrin Hybrids Linked with Dipyrrin, Bidipyrrin or Thiacorrole

**DOI:** 10.3390/molecules22091400

**Published:** 2017-08-23

**Authors:** Renbao He, Huan Yue, Jiahui Kong

**Affiliations:** Zhejiang Yongtai Technology Co. Ltd., Taizhou 317016, China; Renb755701@163.com (R.H.); helenyue668@163.com (H.Y.)

**Keywords:** porphyrin, hybrid, thiacorrole

## Abstract

Novel meso-meso directly linked porphyrin hybrids were successfully targeted and synthesized, with porphyrin units linked with dipyrrin, bidipyrrin or thiacorrole, expanding the ranges of dipyrrin derivatives and showing diverse metal coordinations and further influencing the chemical shift of pyrrole units. The porphyrinyl dipyrrin nickel complex **3** was successfully obtained in a high yield by the oxidation of porphyrinyl dipyrromethane **2** and subsequent coordination. Further oxidative coupling reactions of **3** afforded por-bidipyrrin-por hybrid **4**. Interestingly, an unexpected methoxy por-bidipyrrin-por hybrid **6** was generated by treating **4** with FeCl_3_ in CH_2_Cl_2_/MeOH and subsequent coordination. In addition to open chain hybrids, an aromatic scaffold hybrid por-thiacorrole-por **8** was synthesized by treating porphyrinyl dibromo-dipyrrin nickel complex **7** with Na_2_S·9H_2_O. A series of porphyrin hybrids offers a new approach for π-conjugated molecules.

## 1. Introduction

In recent years, molecules with highly extended π-conjugation have attracted much attention, owing to their excellent properties in organic semiconductor devices, near-infra-red (NIR) dyes and non-linear optical materials [1,2,3,4]. In this regard, dipyrrin, containing conjugated pyrrole units, has been used extensively for synthesis of π-conjugated molecules and fluorescent or colorimetric ion probes [5,6,7,8,9,10,11,12]. Based on dipyrrin, bidipyrrin, which represents a class of bile pigments with two dipyrrin units linked directly by α-carbons, further extends the π-conjugation, and has been extensively researched with regard to catalysis, magnetism and optics [13,14,15,16]. Moreover, covalently linked dipyrrins or bidipyrrins demonstrate a novel approach to obtaining more highly conjugated or more structurally interesting molecules [17,18,19].

**Scheme 1:**
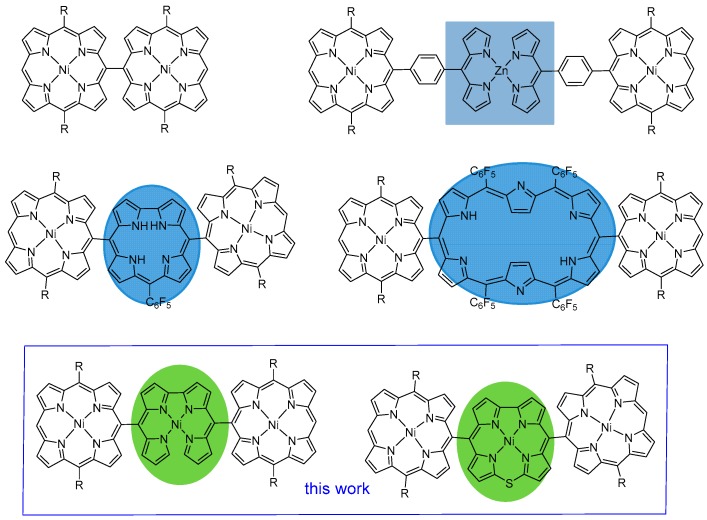
Porphyrin dimer and hybrids linked in various fashions.

On the other hand, porphyrin, a highly conjugated tetrapyrrolic compound, plays an important role in π-extended dyes [20,21,22,23,24]. Various porphyrin types have been explored, including two-dimensionally expanded porphyrin sheets, donor-acceptor type and electron-deficient type (Scheme 1) [25,26,27,28,29]. π-conjugated porphyrins contain multiple porphyrin units linked in various linking fashions, such as meso-meso, β-β, and β-β triply-linked methods [22,30,31,32,33,34]. Among these conjugated porphyrins, *meso-meso* linked porphyrin hybrids with a short center-to-center distance are extremely important, because they are favorable for achieving rapid energy-transfer [35,36]. Additionally, covalently linked multiporphyrin hybrids have different photodynamic and electrochemical properties depending on the type of linkages. For example, the Osuka group has successfully constructed meso-meso linked porphyrin arrays with excellent photophysical properties using a unique oxidation method [37]. Corrole, a ring contracted porphyrinoid, has one peripheral meso carbon less and one inner NH more, compared with porphyrin, which presents different properties for stabilizing higher oxidation states of metals, and a different coordination chemistry. When corrole and porphyrin are linked by the meso-meso method, the hybrids demonstrate novel properties with higher quantum yield and longer life time [38,39]. Moreover, hexaphyrin, an expanded porphyrin type, shows a larger π-conjugation and intra-annular cavity. The meso-meso linked porphyrin-hexaphyrin-porphyrin hybrid revealed a distorted conformation and adopted an Möbius aromatic twisted structure for the [28-π] hexaphyrin moiety in solution [40,41].

Furthermore, when porphyrin and dipyrrin or bidipyrrin are covalently linked, the new dyes simultaneously show the rigid and planar properties of porphyrin unit and the coordination and flexible properties of the dipyrrin or bidipyrrin unit [42,43]. On the other hand, apart from the above-mentioned hybrids, thiacorrole [44], a *meso*-modified porphyrin analogue with heteroatom directly on the π-conjugation circuit, could potentially be introduced into the porphyrin hybrids; this hybrid remains unexplored. The new hybrids could possibly present interesting coordination or magnetism properties related to different units. In spite of rich examples of porphyrin hybrids, to the best of our knowledge, the study of porphyrin dipyrrin, bidipyrrin or thiacorrole hybrids is still rare. Therefore, there is plenty of room for improvement in synthetic routes to the development of new porphyrin hybrids. Moreover, the new hybrids would demonstrate different metal distances and metal coordination environments, and the different environments would effectively influence properties of pyrrole units in the middle part of the hybrids. In the present work, we report the first synthesis of meso-meso directly linked por-dipyrrin-por, por-bidipyrrin-por and por-thiacor-por hybrids, and further investigate the spectroscopic properties of these hybrids.

## 2. Results and Discussion

**Scheme 2:**
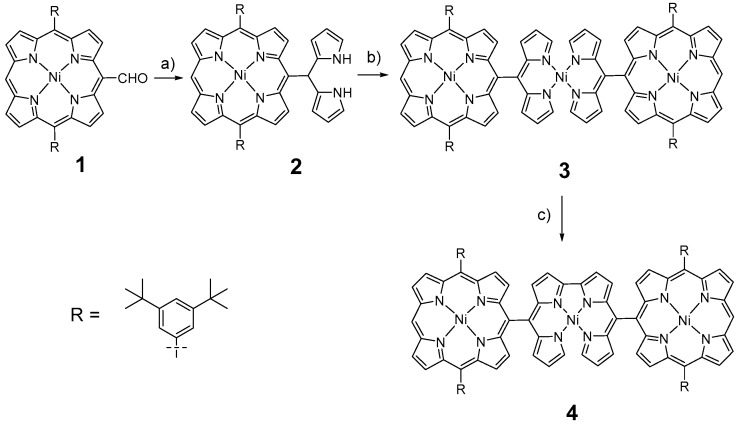
Synthesis of porphyrin dipyrrin hybrid **3** and bidipyrrin hybrid **4**. Reaction conditions: (**a**) excess pyrrole, TFA; (**b**) DDQ, Et_3_N, NiCl_2_·6H_2_O, CH_2_Cl_2_/MeOH; (**c**) DDQ, toluene, reflux.

**Figure 1:**
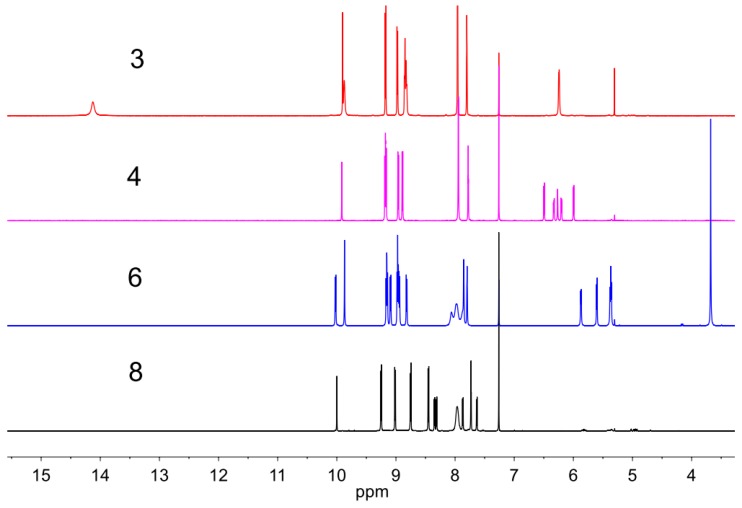
The aromatic regions ^1^H-NMR of porphyrin hybrids **3**, **4**, **6** and **8**.

**Figure 2:**
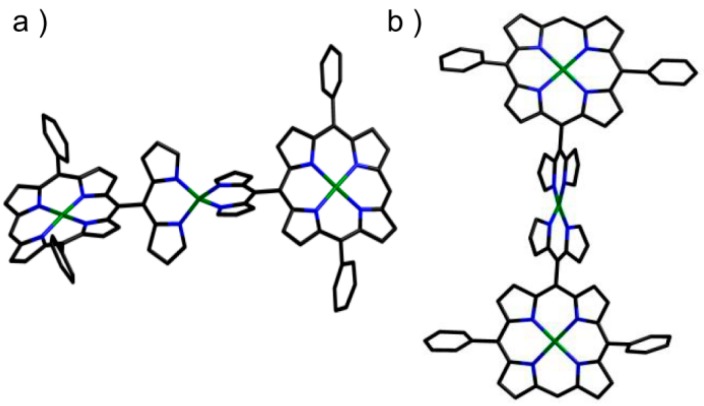
X-ray crystal structures of **3**. Solvent molecules, *tert*-butyl groups and hydrogen atoms were omitted for clarity (**a**) top view; (**b**) side view.

**Figure 3:**
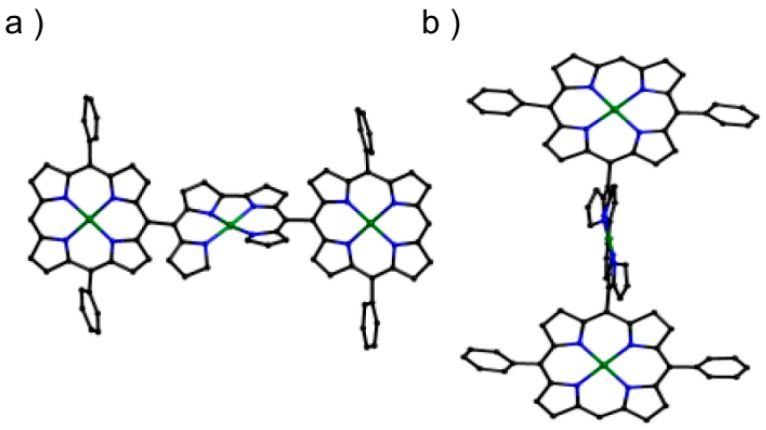
X-ray crystal structures of **4**. Solvent molecules, *tert*-butyl groups and hydrogen atoms were omitted for clarity (**a**) top view; (**b**) side view.

A synthetic route for porphyrin-dipyrrin-porphyrin (por-dipyrrin-por) hybrid was achieved by following the method illustrated in Scheme 2. *meso*-Porphyrinyl dipyrromethane **2** was prepared by TFA-catalyzed condensation of *meso*-formylated Ni(II) porphyrin **1** with excess pyrrole in a 65% yield. After dipyrromethane **2** was oxidized with 2,3-dichloro-5,6-dicyano-1,4-benzoquinone(DDQ) in THF, and reacted with NiCl_2_·6H_2_O, the porphyrinyl dipyrrin nickel complex (por-dipyrrin-por) **3** was obtained in a good yield. The molecular mass of the triad, detected by HRMS, was fully consistent with calculated value: *m*/*z* [M + H]^+^ = 1825.7453 (calcd. for 1825.7428). The ^1^H-NMR spectrum of **3** in CDCl_3_ revealed that as the result of the electron withdrawing and paramagnetism effect of nickel metal and deshielding influences of aromatic circulation of the two near porphyrin cycle, the broad signals for four pyrrolic α-CHs and pyrrolic β-CHs in dipyrrin units were downfielded and observed at 13.96 ppm and 9.89 ppm, respectively (Figure 1). Because of the effect of nickel metal, the remaining α positions of pyrroles become more reactive, and could potentially be oxidized to form a new C-C bond. Consistent with this, when complex **3** was reacted with DDQ in reflux toluene, the dipyrrin units were successfully linked with a new C-C bond to afford a novel por-bidipyrrin-por hybrid **4** in a 59% yield. HRMS of **4** lied in *m/z* [M + H]^+^ = 1823.7160, which is consistent with calcd. result (calcd. for 1823.7272). The ^1^H-NMR spectrum of **4** in CDCl_3_ confirmed the structure with the disappearance of the downfield broad signal at 13.96 ppm, and the pyrroles CHs appeared at 5.9–6.7 ppm. It is worth noting that, compared with hybrid **3**, the α-CHs of **4** was significantly upfielded owing to the C-C bond, which is consistent with the reported results [13,14,15,16]. More solid structural evidence for **3** and **4** came from the single crystal X-ray diffraction analyses (Figure 2 and Figure 3), which revealed that the Ni^II^ ion of complex **3** is coordinated with two dipyrrin chelates in a pseudo-tetrahedral environment, as expected for such compound. However, the Ni^II^ ion adopted a distorted square planar environment for complex **4**, as the result of the repulsion between the two remaining α-CHs.

**Scheme 3:**
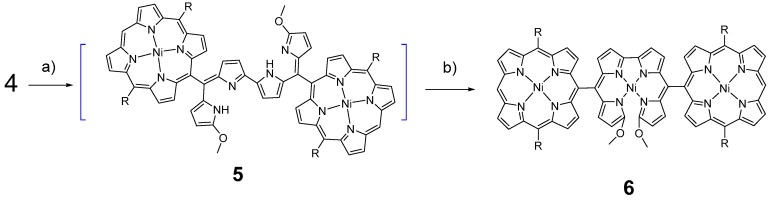
Synthesis of substituted porphyrin bidipyrrin hybrid **6**. Reaction conditions: (**a**) FeCl_3_, CH_2_Cl_2_/MeOH; (**b**) Ni(OAc)_2_·2H_2_O, CH_2_Cl_2_/MeOH.

On the other hand, hybrid **4** demonstrates the porphyrin linked by an opened tetrapyrrolic complex. We wanted to explore the possibility to further synthesizing hybrids linked by macrocyclic frameworks. Considering that there are α positions remaining in **4**, it is possible to form further conjugated frameworks, when exposed to some oxidants. Interestingly, when **4** was treated with FeCl_3_ in CH_2_Cl_2_/MeOH, a new product (**5**) was detected (Scheme 3). Unfortunately, compound **5** was unstable. We used Ni(OAc)_2_·2H_2_O to coordinate with **5** to afford complex **6**. The ^1^H-NMR spectrum of **6** revealed that the signal of methoxy groups appeared at 3.69 ppm, indicating no expected ring-closure products. Compared with **4**, the β-CHs of the bidipyrrin unit in **6** were slightly upfielded, which may be ascribed to the electron-donating effect of the methoxy groups.

**Scheme 4:**
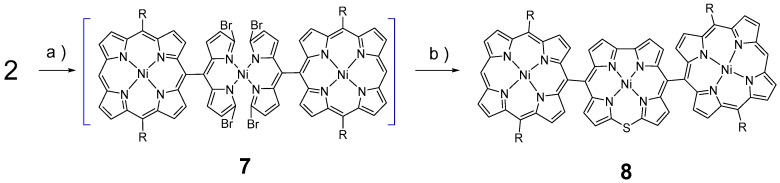
Synthesis of porphyrin thiacorrole hybrid **8**. Reaction conditions: (**a**) (i) NBS, −78 °C; (ii) DDQ; (iii) NiCl_2_·6H_2_O, THF/MeOH; (**b**) Na_2_S·9H_2_O, DMF.

In addition to the open chain units, it is interesting to investigate porphyrin hybrids linked with aromatic scaffolds. The synthetic protocol of por-thiacorrole-por hybrids was illustrated in Scheme 4. Consecutive treatment of porphyrinyldipyrromethane **2** with NBS, DDQ and NiCl_2_·6H_2_O gave the bis(dibromodipyrrin)Ni(II) complex **7** in a high yield (by TLC). Unfortunately, complex **7** was unexpectedly unstable, and was able to be decomposed when exposed to the air. We found that the 10-thiacorrole **8** was obtained by treatment of complex **8** with sodium sulfide hydrate in DMF at 65 °C in an excellent yield. The ^1^H-NMR spectrum of hybrid **8** revealed that the β-CHs in porphyrin units lay in the range of 8.4–9.4 ppm, and the meso-CHs lay at 10.0 ppm, which is consistent with porphyrin properties. Interestingly, the β-CHs of the 10-thiacorrole unit lay in the range of 7.6–8.4 ppm, slightly downfielded compared with **4**, which is related to the aromatic scaffold.

**Figure 4:**
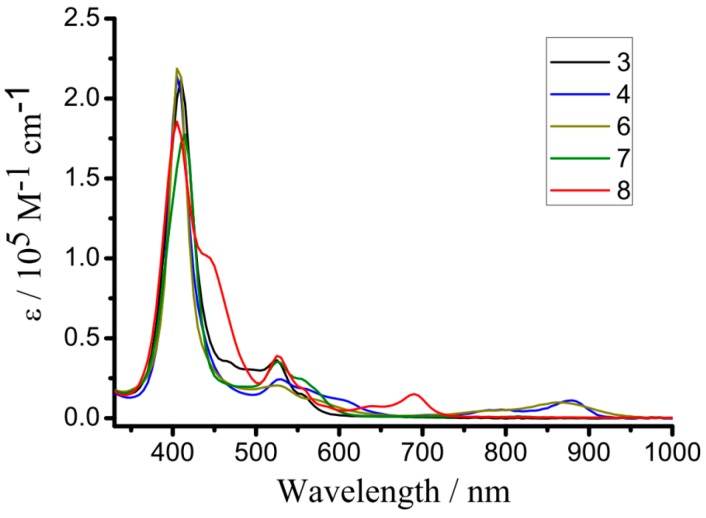
The absorption spectra of **3**, **4**, **6**, **7** and **8** in CH_2_Cl_2_.

The absorption spectra of porphyrin hybrid compounds are shown in Figure 4. Porphyrin-dipyrrin complex **3** shows a sharp Soret peak at 410 nm and weak peak at 530 nm, which is similar to the spectra addition of porphyrin and dipyrrin, indicating less electron transfer between porphyrin and dipyrrin unit. This phenomenon could be ascribed to the approximately vertical dihedral angle between two units in the crystal. Compared with **3**, por-bidipyrrin-por hybrid **4** presents a similar S band at 410 nm, shoulder peaks around 544 nm and a bathochromic-shift peak at 880 nm, which may be related to the conjugated bidipyrrin unit in the NIR component. Methoxy hybrid 6 shows a similar S bond with **4**, with a slight hypochromatic shifted Q bond at 860 nm. Porphyrin-dibromodipyrrin complex **7** shows an analogous spectrum to **3**, owing to their similar structures. In contrast to the open chain hybrids, compound **8** shows a subdued but broad S band at 410 nm, slightly red-shifted shoulder peaks at 540 nm and a broad Q band at 690 nm, which could be related to the thiacorrole unit.

## 3. Materials and Synthesis

### 3.1. Materials and Instrumentation

All the reactions were performed in oven-dried reaction vessels under N_2_. Commercially available solvents and reagents were used without further purification unless otherwise mentioned. Thin-layer chromatography (TLC) was carried out on aluminum sheets coated with silica gel 60 F254 (MERCK, Darmstadt, Germany). ^1^H-NMR spectra were obtained using a Bruker (Karlsruhe, Germany) AM 400 spectrometer, and chemical shifts were reported relative to CDCl_3_ (*δ* = 7.26) in ppm. ^13^C-NMR spectra were recorded at 100 MHz (Bruker AM 400) and chemical shifts were reported relative to CDCl_3_ (*δ* = 77.00) in ppm. HRMS were performed using a Waters LCT Premier XE spectrometer (Milford, MA, USA). UV-Vis absorption spectra were recorded on a Varian Cary 100 spectrophotometer and fluorescence spectra were recorded on a Varian Cary Eclipse (Anaheim, CA, USA) fluorescence spectrophotometer.

### 3.2. Crystallography

X-ray analyses were performed on a SMART APEX equipped with CCD detector (Bruker, Karlsruhe, Germany) using MoKα (graphite, monochromated, λ = 0.71069 Å) radiation and CuKα (graphite, monochromated, λ = 1.54178 Å) radiation. The structure was solved by the direct method of SHELXS-97 and refined using the SHELXL-97 program. The positional parameters and thermal parameters of non-hydrogen atoms were refined anisotropically on F^2^ by the full-matrix least-squares method. Hydrogen atoms were placed at calculated positions and refined riding on their corresponding carbon atoms (Table 1). Single crystals of **3** and **4** were obtained by the slow recrystallization from solutions in dichloromethane/methanol.

### 3.3. Syntheses

#### 3.3.1. Synthesis of **2**

Trifluoroacetic acid (0.25 mL, 2.6 mmol) was added to a solution of Ni^II^ (10,20-di(3,5-di-tert-butylphenyl))-5-formyl porphyrin (2.0 g, 2.6 mmol) and pyrrole (36 mL, 0.52 mol) and stirred for 2 h at room temperature under nitrogen. After neutralization with 1 N NaOH aq. (3.0 mL), the solution was extracted with CH_2_Cl_2_, dried over anhydrous Na_2_SO_4_ and evaporated in vacuo. Unreacted pyrrole was distilled under vacuum and the residue was purified by silica-gel column chromatography with a mixture of CH_2_Cl_2_ and n-hexane (*v/v* = 1/1) as an eluent. Recrystallization from CH_2_Cl_2_/MeOH gave red solid **2** (1.4 g, 65% yield).

^1^H-NMR (400 MHz, CDCl_3_) δ 9.71 (s, 1H), 9.20 (d, *J* = 5.2 Hz, 2H), 9.08 (d, *J* = 4.8 Hz, 2H), 8.88 (d, *J* = 4.8 Hz, 2H), 8.78 (d, *J* = 5.2 Hz, 2H), 8.11 (s, 2H), 7.83 (d, *J* = 1.8 Hz, 4H), 7.77 (s, 1H), 7.74 (t, *J* = 1.8 Hz, 2H), 6.62 (dd, *J* = 4.2, 2.6 Hz, 2H), 6.32 (d, *J* = 3.6 Hz, 2H), 6.24 (dd, *J* = 6.0, 2.8 Hz, 2H), 1.49 (s, 37H). ^13^C-NMR (100 MHz, CDCl_3_) δ 149.05, 142.63, 142.33, 142.01, 141.74, 139.44, 135.16, 133.64, 132.93, 132.29, 130.27, 128.60, 121.26, 119.54, 116.99, 108.54, 107.34, 104.56, 43.45, 34.98, 31.67. HRMS [M + H]^+^ *m/z* = 887.4242, calcd. for C_57_H_61_N_6_Ni = 887.4311.

#### 3.3.2. Synthesis of **3**

A solution of **2** (1.0 g, 1.12 mmol) in THF (250 mL) was treated with DDQ (280 mg, 1.24 mmol) at room temperature. After 50 min, 1 mL of Et_3_N were added. A methanolic solution containing NiCl_2_·6H_2_O (2.0 g in 30 mL) was then added to the reaction mixture. The reaction proceeded for an additional 45 min, after which the TLC indicated no uncoordinated dipyrromethene. The reaction mixture was washed with water, dried over anhydrous Na_2_SO_4_ and evaporated in vacuo. The residue was purified by silica-gel column chromatography with CH_2_Cl_2_ as an eluent. Recrystallization from CH_2_Cl_2_/MeOH gave the deep red solid **3** (810 mg, 79% yield).

^1^H-NMR (400 MHz, CDCl_3_) δ 14.12 (s, 4H), 9.90 (s, 2H), 9.87 (s, 4H), 9.18 (d, *J* = 4.8 Hz, 4H), 8.98 (d, *J* = 4.8 Hz, 4H), 8.84 (dd, *J* = 11.8, 4.4 Hz, 8H), 7.96 (m, 8H), 7.80 (s, 4H), 6.24 (d, *J* = 2.8 Hz, 4H), 1.55 (s, 72H). ^13^C-NMR (100 MHz, CDCl_3_) δ 150.00, 148.98, 143.41, 142.74, 142.51, 142.13, 139.88, 132.93, 132.66, 131.96, 128.84, 121.20, 120.29, 110.24, 105.52, 35.03, 31.71. HRMS [M + H]^+^ *m/z* = 1825.7453, calcd. for C_114_H_114_N_12_Ni_3_ = 1825.7428.

#### 3.3.3. Synthesis of **4**

A solution of **3** (1.1 g, 0.63 mmol) in toluene (150 mL) was treated with DDQ (0.21 g, 0.91 mmol) and heated at reflux for 12 h. After removal of the solvent, the residue was separated by silica gel column chromatography using CH_2_Cl_2_/hexane = 1/2 (*v/v*) to afford **4** as a deep brown solid (650 mg, 59% yield).

^1^H-NMR (400 MHz, CDCl_3_) δ 9.92 (s, 2H), 9.18 (dd, *J* = 6.0, 5.0 Hz, 8H), 8.96 (d, *J* = 4.8 Hz, 4H), 8.89 (d, *J* = 4.8 Hz, 4H), 7.94 (m, 8H), 7.78 (m, 4H), 6.50 (d, *J* = 4.4 Hz, 2H), 6.32 (s, 2H), 6.27 (s, 2H), 6.21 (s, 2H), 6.00 (d, *J* = 4.4 Hz, 2H), 1.52 (s, 72H). ^13^C-NMR (100 MHz, CDCl_3_) δ 161.76, 153.19, 149.07, 144.82, 143.53, 143.28, 142.95, 142.64, 141.95, 140.97, 139.96, 133.11, 132.78, 132.22, 128.91, 121.31, 120.49, 116.16, 111.46, 105.59, 35.10, 31.79. HRMS [M + H]^+^ *m/z* = 1823.7160, calcd. for C_114_H_113_N_12_Ni_3_ = 1823.7272.

#### 3.3.4. Synthesis of **5**

A methanol solution (30 mL) of FeCl_3_ (50 mg, 0.32 mmol) was added to a solution of **4** (110 mg, 0.06 mmol) in CH_2_Cl_2_ (100 mL). After stirring for 4 h, the reaction mixture was washed with water. The organic phase was dried over sodium sulfate, filtered, and concentrated under reduced pressure. The crude product (**5**) was used for the next reaction without purification.

#### 3.3.5. Synthesis of **6**

An ethanol (20 mL) solution of Ni(OAc)_2_·2H_2_O (100 mg, 0.44 mmol) was added to a toluene (70 mL) solution of **5** (110 mg, 0.06 mmol). The mixture was stirred at 70 °C for 4 h. After evaporation under vacuum, the residue was separated by silica gel column chromatography using CH_2_Cl_2_/hexane (1/2, *v/v*) to afford complex **6** (86 mg, 72%) as a dark red solid.

^1^H-NMR (400 MHz, CDCl_3_) δ 10.02 (d, *J* = 4.8 Hz, 2H), 9.87 (s, 2H), 9.19–9.13 (m, 4H), 9.09 (d, *J* = 4.8 Hz, 2H), 9.00–8.91 (m, 6H), 8.82 (d, *J* = 4.8 Hz, 2H), 7.92 (m, 12H), 5.87 (d, *J* = 4.0 Hz, 2H), 5.60 (d, *J* = 4.8 Hz, 2H), 5.44–5.32 (m, 4H), 3.68 (s, 6H), 1.57 (s, 72H). ^13^C-NMR (100 MHz, CDCl_3_) δ 177.37, 150.50, 149.01, 143.57, 142.82, 142.47, 142.24, 140.53, 139.86, 138.03, 133.03, 132.58, 132.43, 131.64, 128.78, 127.84, 121.28, 120.05, 112.47, 111.31, 105.39, 103.84, 35.11, 31.81. HRMS [M + H]^+^ *m/z* = 1883.7591, calcd. for C_116_H_117_N_12_Ni_3_O_2_ = 1883.7483.

#### 3.3.6. Synthesis of **7**

A solution of **2** (1.0 g, 1.12 mmol) in dry THF (250 mL) was cooled to −78 °C and NBS (400 mg, 2.24 mmol) and added in two portions over 1 h. A THF solution of DDQ (280 mg, 1.24 mmol) was added dropwise over 10 min. The reaction mixture was warmed to room temperature. After 50 min, 1 mL of Et_3_N was added. A methanolic solution containing NiCl_2_·6H_2_O (2.0 g in 30 mL) was then added to the reaction mixture. The reaction proceeded for an additional 45 min. The reaction mixture was washed with water, dried over anhydrous Na_2_SO_4_, and evaporated in vacuo. The residue was purified by fast silica-gel column chromatography with CH_2_Cl_2_ as an eluent to give the deep red solid **7** (810 mg, 79% yield). The crude product was immediately used for the next reaction. HRMS *m/z* = 2137.3844, calcd. for C_114_H_111_Br_4_N_12_Ni_3_ = 2137.3849.

#### 3.3.7. Synthesis of **8**

A Schlenk tube containing **7** (122.2 mg, 50 mmol) and Na_2_S·9H_2_O (24.0 mg, 0.10 mmol) was evacuated and then refilled with N_2_. Dry DMF (15 mL) was added, and mixturewas stirred at 65 °C for 5 h in oil bath. After cooling the reaction mixture to RT, The reaction mixture was washed with water, dried over anhydrous Na_2_SO_4_, and evaporated in vacuo. The mixture was purified by silica-gel column chromatography (CH_2_Cl_2_/hexane) to give **8** (12.6 mg, 20.7 mmol) in a 41% yield as black solid.

^1^H-NMR (400 MHz, CDCl_3_) δ 10.00 (s, 2H), 9.25 (d, *J* = 4.8 Hz, 4H), 9.01 (d, *J* = 4.7 Hz, 4H), 8.75 (d, *J* = 4.9 Hz, 4H), 8.45 (d, *J* = 4.9 Hz, 4H), 8.35 (d, *J* = 4.5 Hz, 2H), 8.31 (d, *J* = 4.7 Hz, 2H), 7.97 (s, 8H), 7.87 (d, *J* = 4.7 Hz, 2H), 7.73 (t, *J* = 1.7 Hz, 4H), 7.63 (d, *J* = 4.5 Hz, 2H), 1.48 (s, 72H).^13^C-NMR (100 MHz, CDCl_3_) δ 148.94, 145.47, 143.59, 142.74, 139.98, 139.51, 133.23, 132.71, 132.11, 130.72, 128.84, 121.65, 121.18, 120.76, 119.01, 105.50, 34.98, 31.66. HRMS [M + H]^+^ *m/z* = 1853.6588, calcd. for C_114_H_111_N_12_Ni_3_S = 1853.6836.

## 4. Conclusions

In summary, we have successfully prepared a series of porphyrin hybrids with variously coordinated nickel metal. By oxidizing meso-Porphyrinyldipyrromethene **2** using DDQ in THF and subsequent coordination, por-dipyrrin-por hybrid **3** was obtained in a high yield. The further oxidization of **3** afforded the por-bidipyrrin-por hybrid **4**. Unexpectedly, treating **4** with FeCl_3_ in CH_2_Cl_2_/MeOH and subsequent coordination generated methoxy-substituted por-bidipyrrin-por hybrid **6**. Fortunately, by treating dibromo por-dipyrrin-por hybrid **7** with Na_2_S·9H_2_O, the expected por-thiacorrole-por hybrid **8** was successfully synthesized. In conclusion, meso-meso linked porphyrin hybrids play an important role in photoelectric or magnetic materials. Various linking methods were proven to be effective approaches for functional porphyrin materials. The further properties of these hybrids will be the main objective of our future work.

## Figures and Tables

**Table 1:** Crystallographic data and structure refinement summary for the compounds.

3	4
C_114_H_114_N_12_Ni_3_	C_114_H_112_N_12_Ni_3_
*Mr* = 1828.30	*Mr* = 1826.29
Orthorhombi	Triclinic
*C222(1)*	*P-1*
*a* = 33.100(3)	*a* = 19.6380(18)
*b* = 24.600(2)	*b* = 20.1721(19)
*c* = 15.4400(14)	*c* = 27.330(3)
*α* = 90.00°	*α* = 73.257(2)°
*β* = 90.00°	*β* = 78.750(3)°
*γ* = 90.00°	*γ* = 67.5910(10)°
*V* = 12572.3(19) Å^3^	*V* = 9540.1(15) Å^3^
*Z* = 4	*Z* = 3
*R_1_* = 0.1257	*R_1_* = 0.1264
*wR_2_* = 0.2693	*wR_2_* = 0.2897
GOF = 1.038	GOF = 1.084

CCDC 1550953 (**3**) and 1550954 (**4**) contain the supplementary crystallographic data for this paper. These data can be obtained free of charge from The Cambridge Crystallographic Data Centre via www.ccdc.cam.ac.uk/data_request/cif.

## Supplementary Materials

Supplementary materials are available online.
