# Supplementary material for: Covalent Porphyrin Hybrids Linked with Dipyrrin, Bidipyrrin or Thiacorrole
Source: Molecules. 2017 Aug 23;22(9):1400. doi: 10.3390/molecules22091400 (PMC6151592; doi:10.3390/molecules22091400)
Supplement: Supplementary file 1 [file molecules-22-01400-s001.pdf]

Supplementary Materials

Covalent Porphyrin Hybrids Linked with Dipyrrin, Bidipyrrin or Thiacorrole

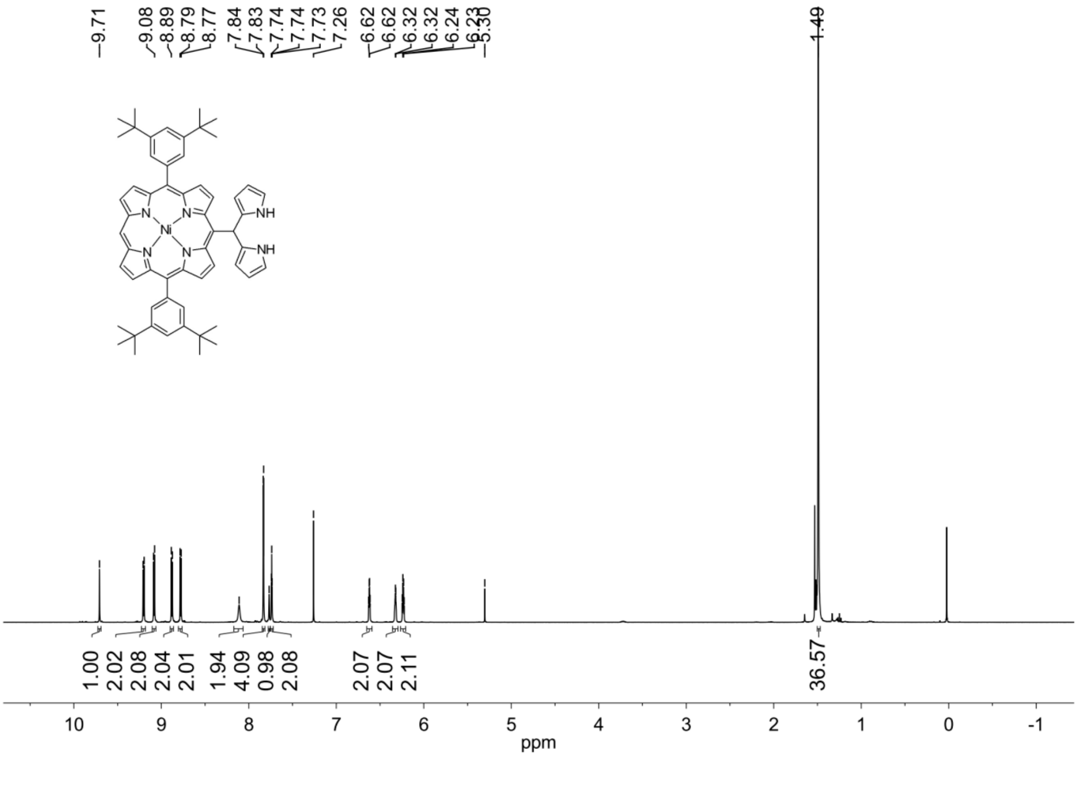

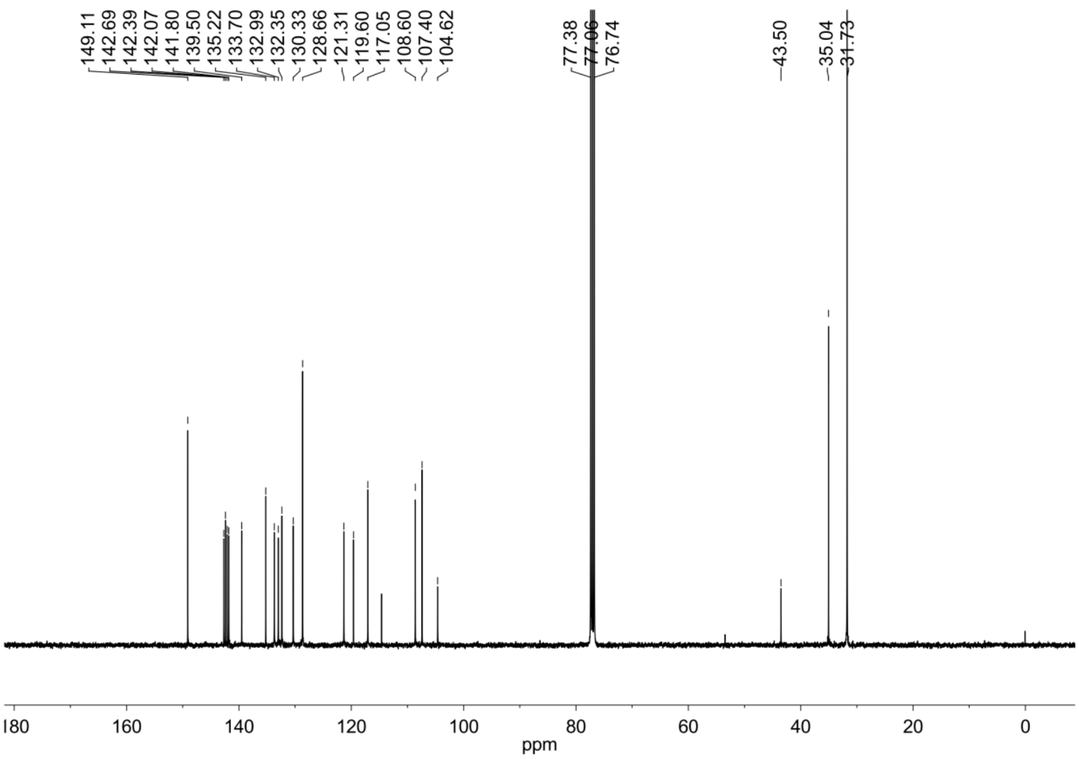

Figure S1. ^{1}H and ^{13}C NMR spectra of **2** in $CDCl_3$.

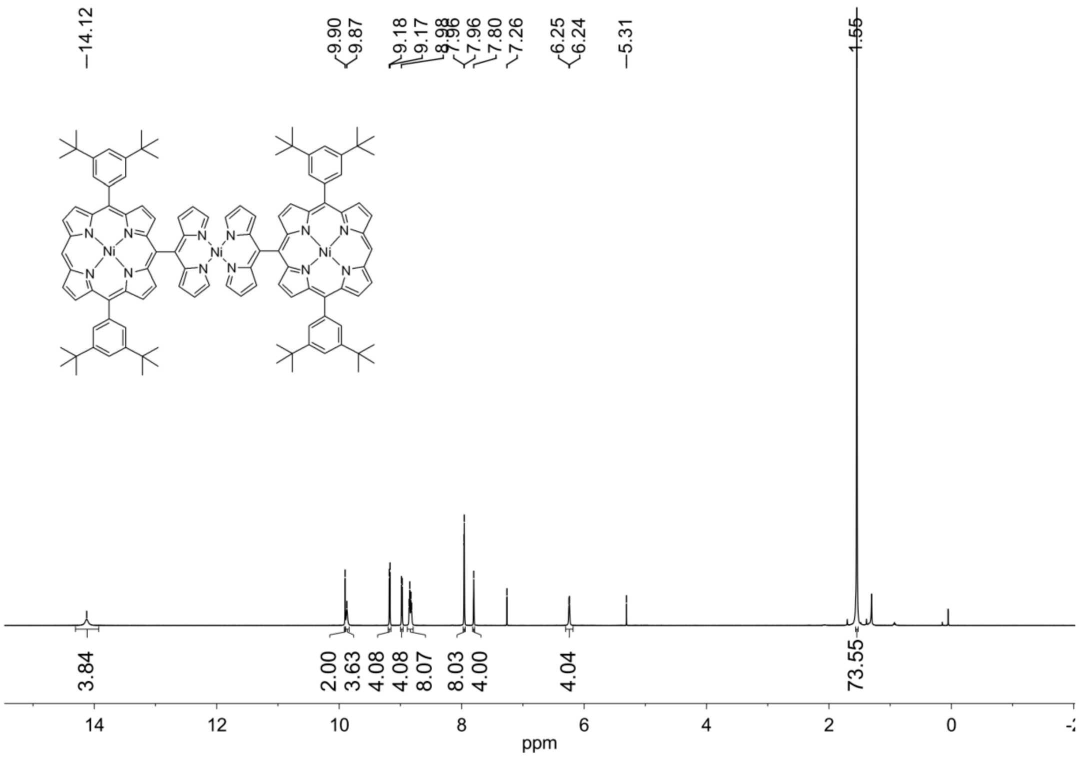

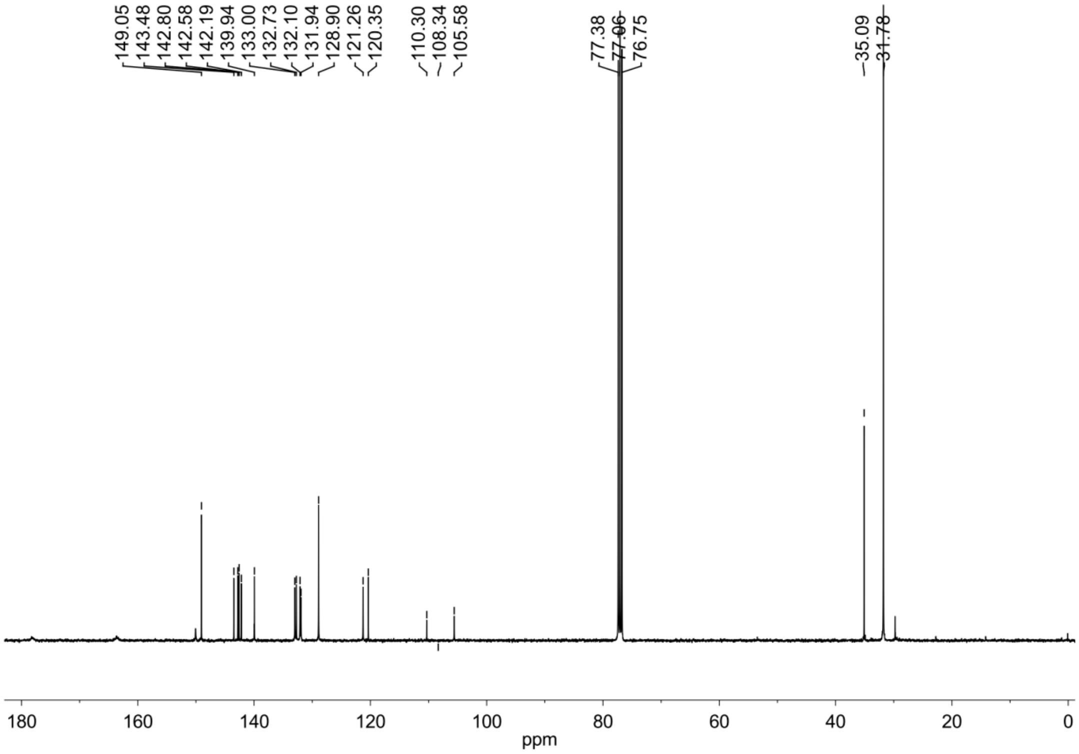

Figure S2. ^{1}H and ^{13}C NMR spectra of **3** in $CDCl_3$.

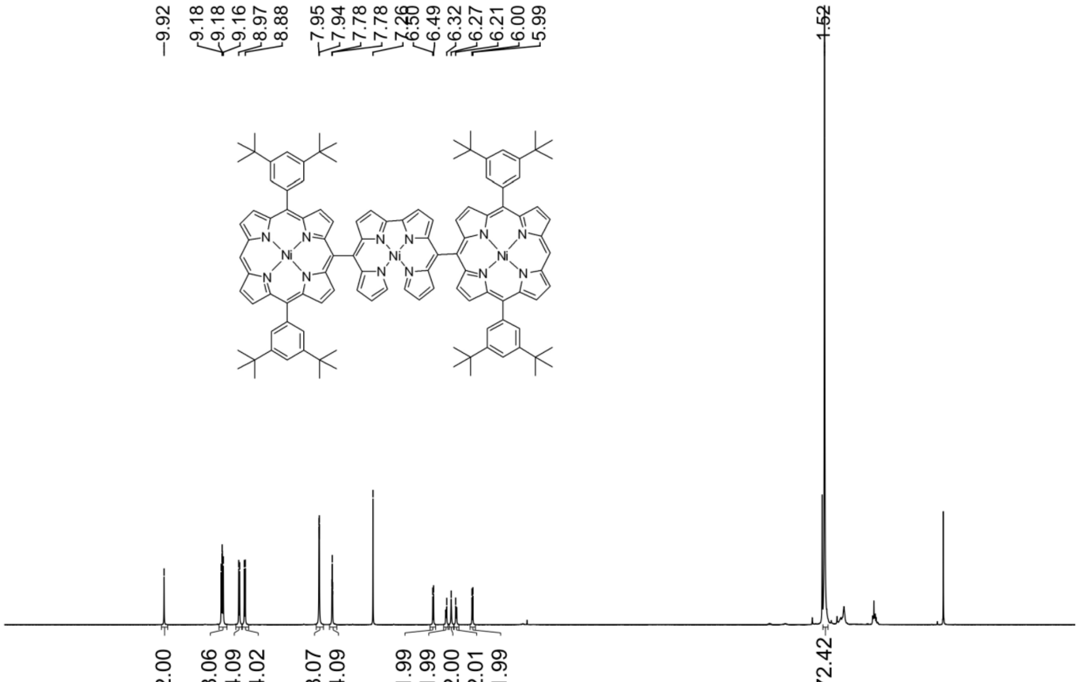

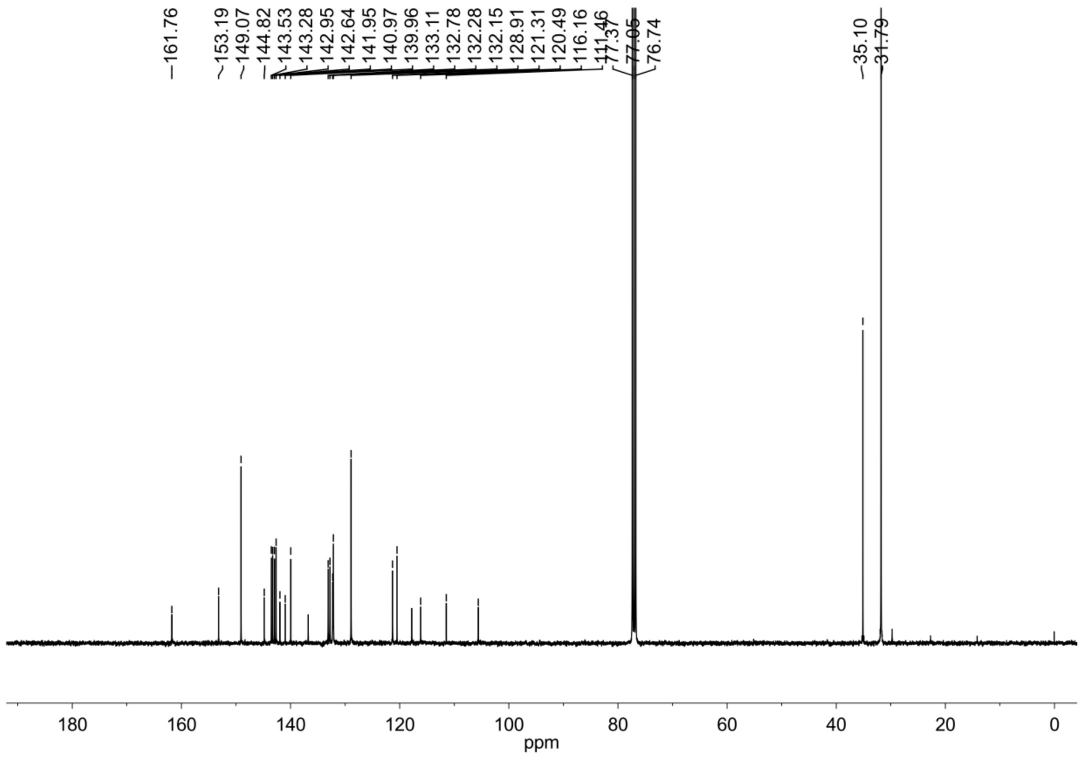

Figure S3. ^{1}H and ^{13}C NMR spectra of **4** in $CDCl_3$.

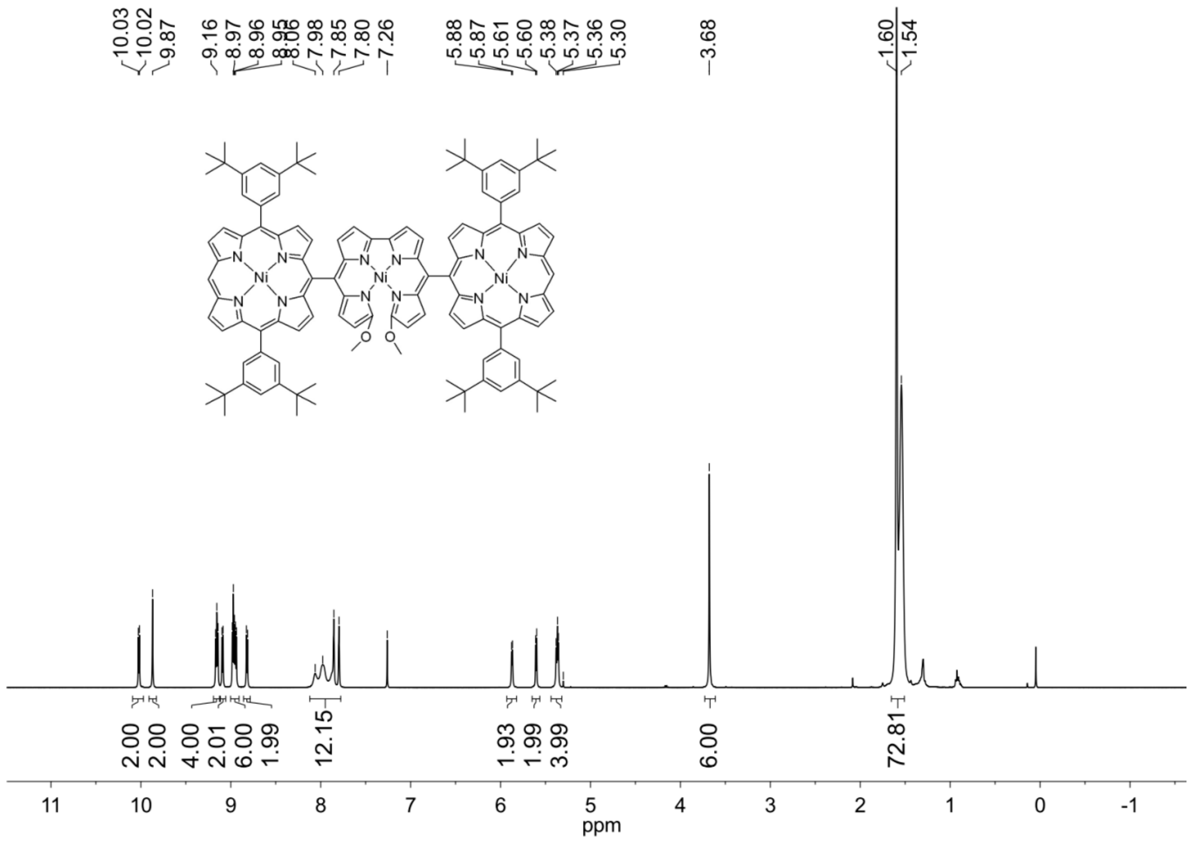

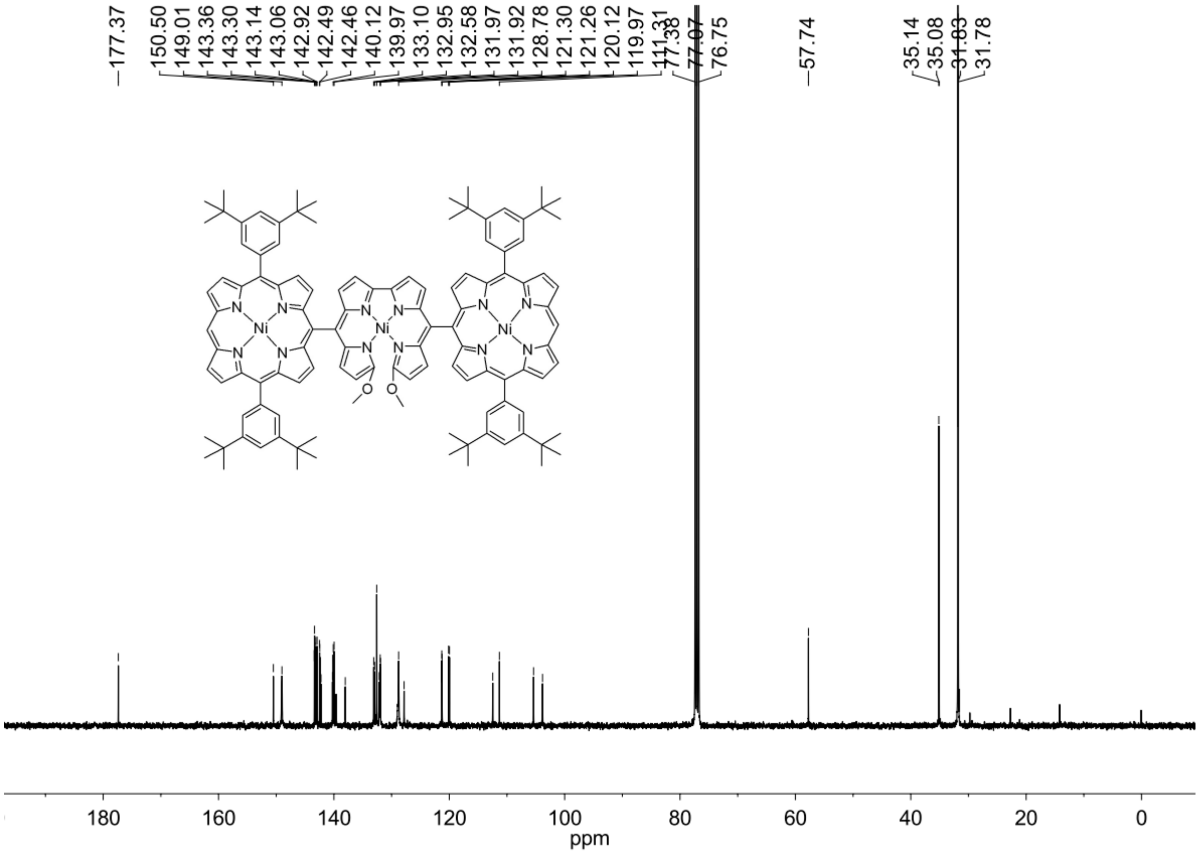

Figure S4. ^{1}H and ^{13}C NMR spectra of **6** in $CDCl_3$.

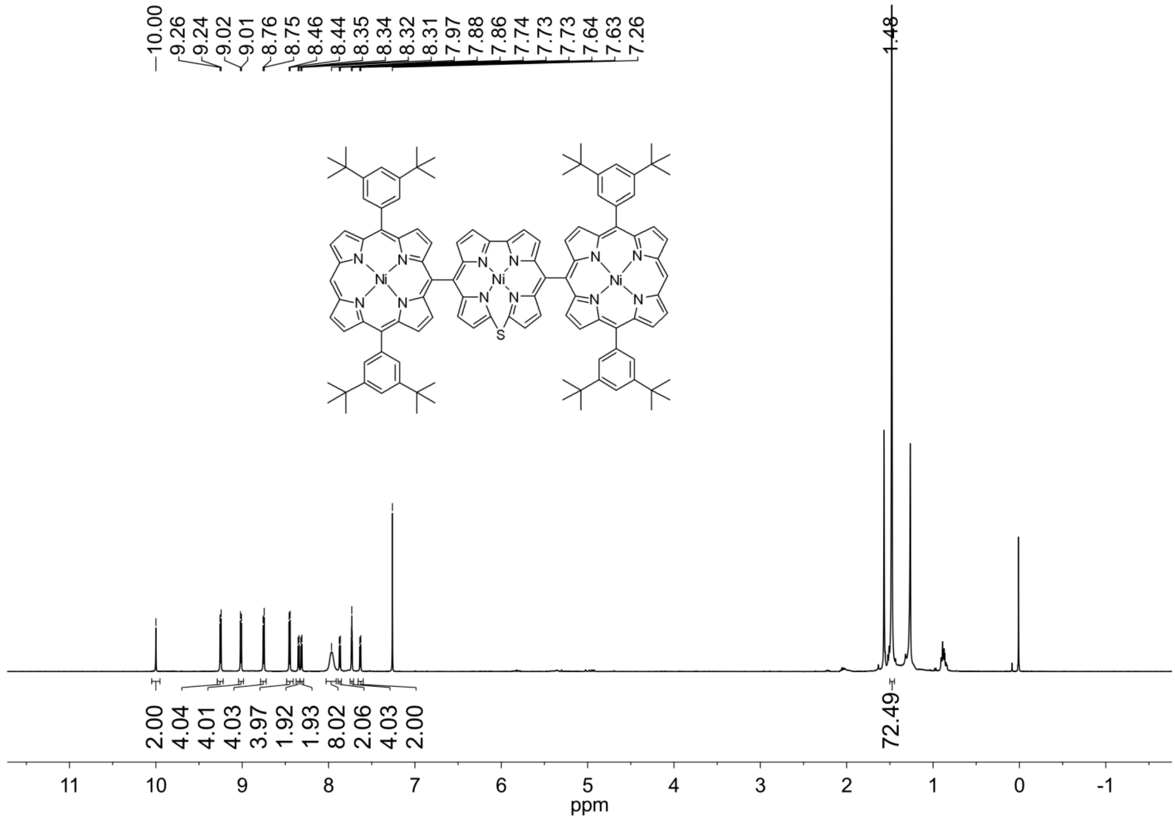

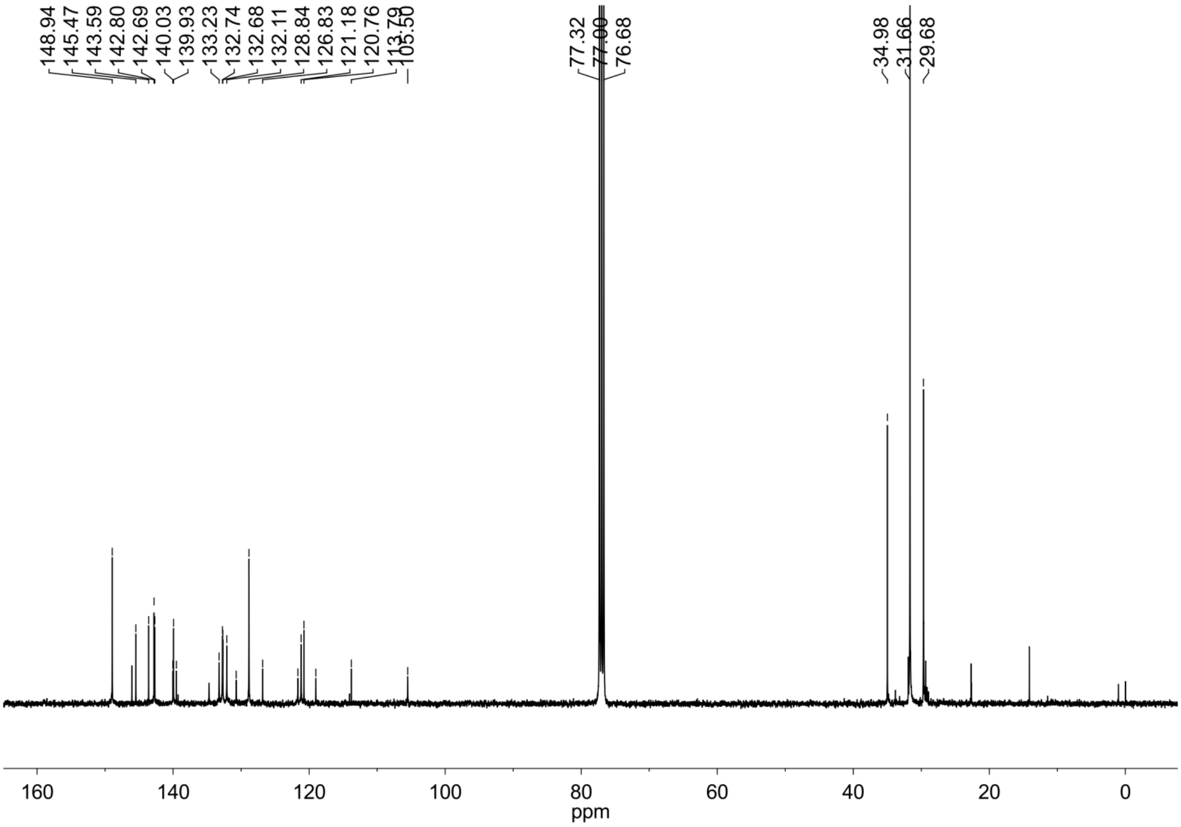

Figure S5. ^{1}H and ^{13}C NMR spectra of **8** in $CDCl_3$.

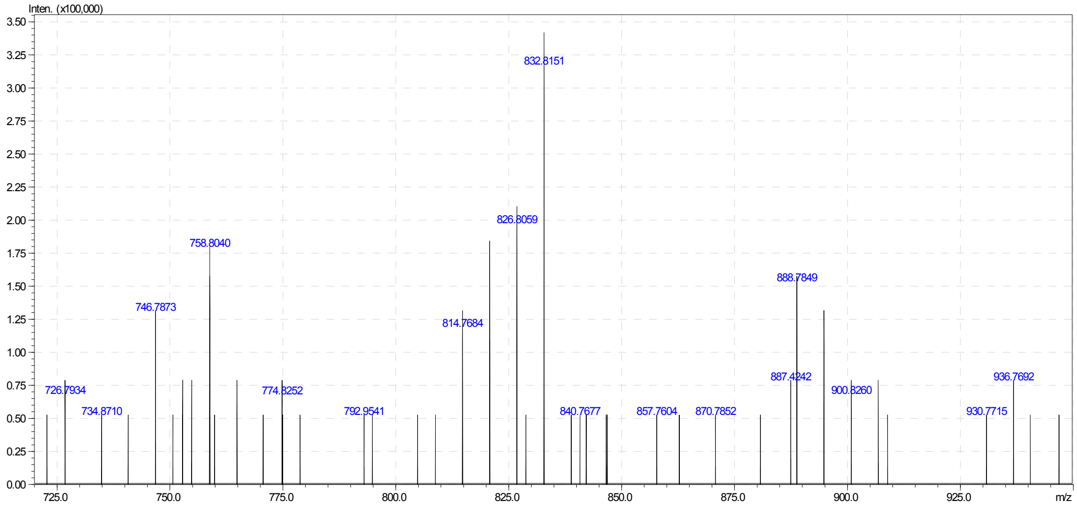

Figure S6. HRMS of **2** in CH_2Cl_2.

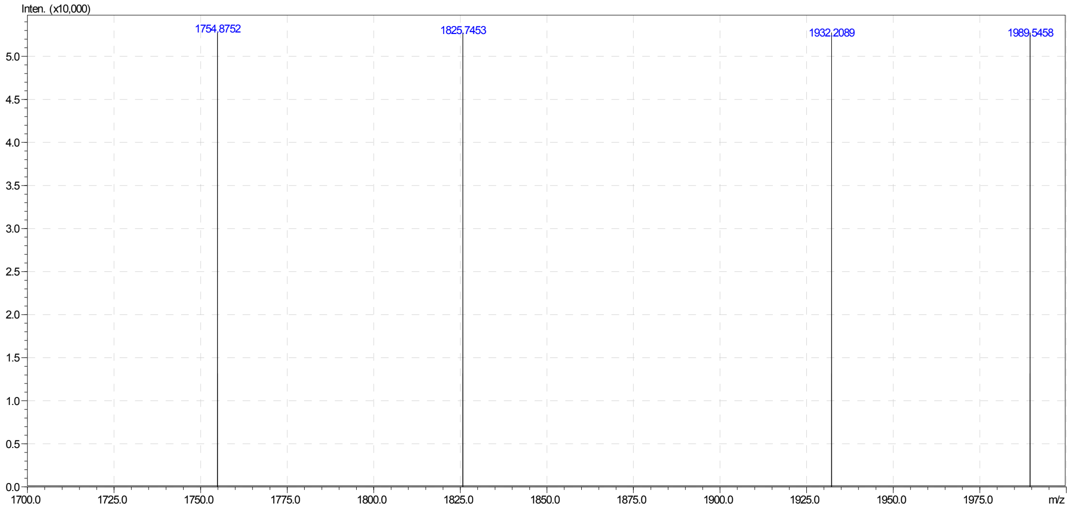

Figure S7. HRMS of **3** in CH_2Cl_2.

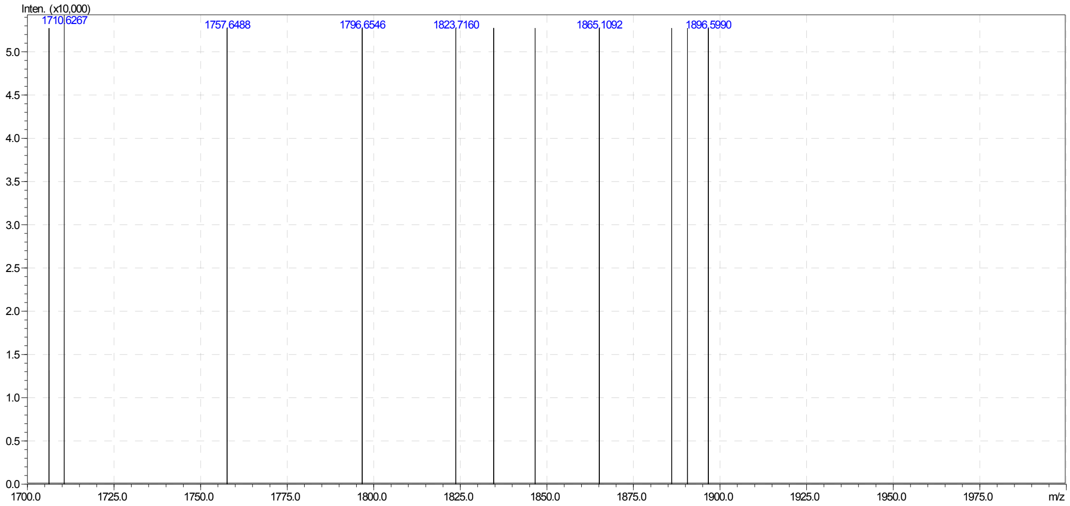

Figure S8. HRMS of **4** in CH_2Cl_2.

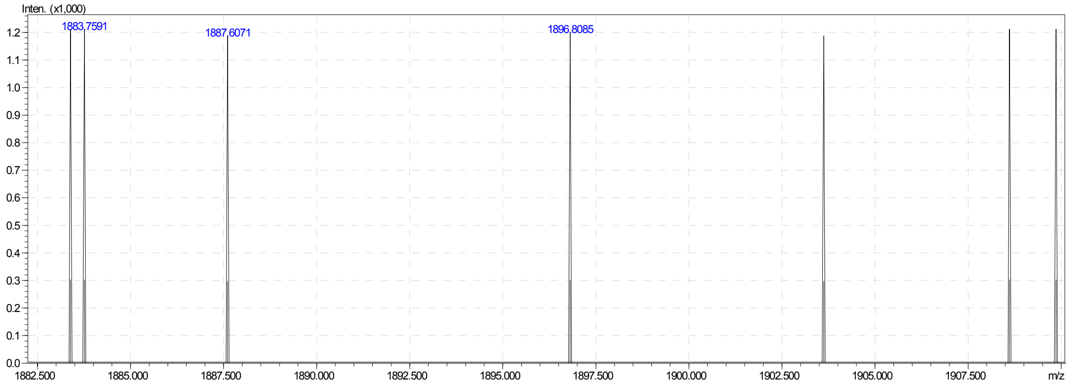

Figure S9. HRMS of **6** in CH_2Cl_2.

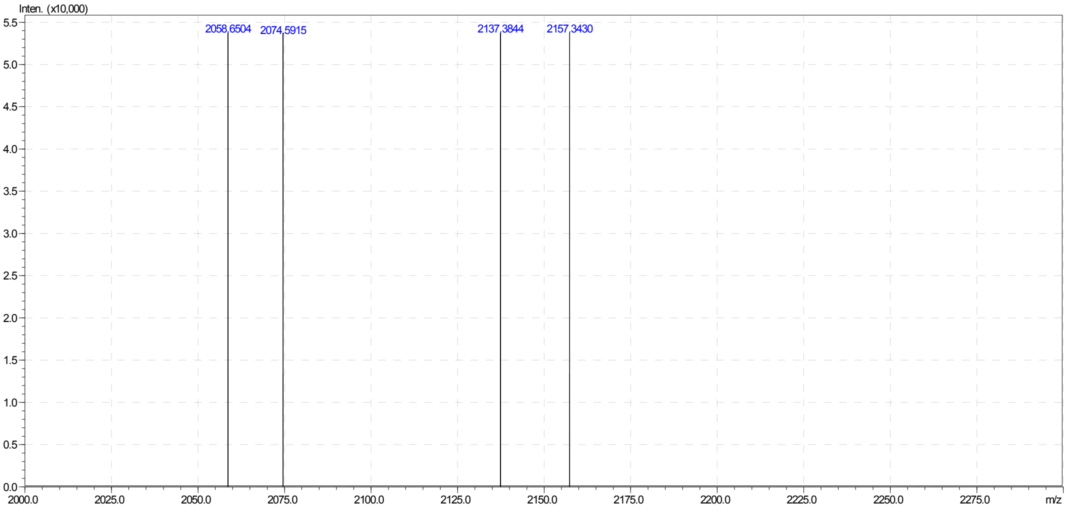

Figure S10. HRMS of **7** in CH_2Cl_2.

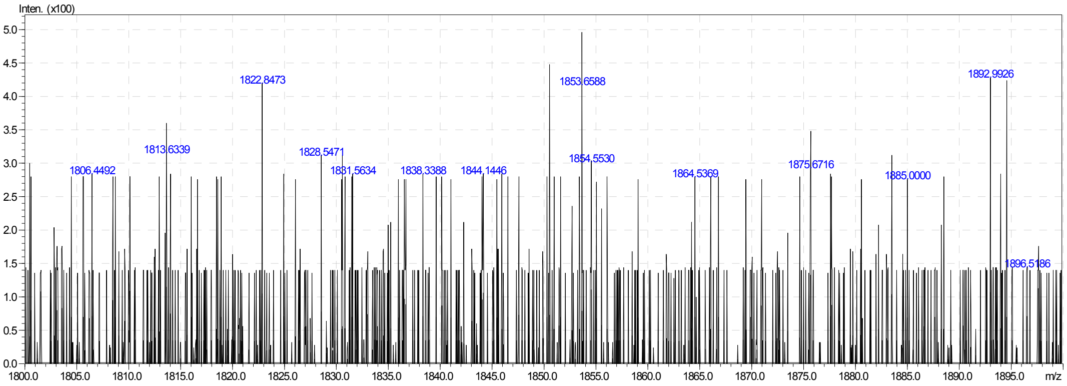

Figure S11. HRMS of **8** in CH_2Cl_2.
